# The roles of intrinsic motivation and capability-related factors in cognitive effort-based decision-making

**DOI:** 10.3389/fpsyg.2024.1303262

**Published:** 2024-05-02

**Authors:** Alyssa Randez, Sébastien Hélie

**Affiliations:** CCN Lab, Department of Psychological Sciences, Purdue University, West Lafayette, IN, United States

**Keywords:** cognitive effort, demand preference, capability judgments, adaptive algorithm, individual differences

## Abstract

Individual differences in cognitive effort-based decision-making can be used to reveal human motivations to invest effort into a given task. Preferences among options that differ by dimensions related to demand levels (i.e., the interaction of task characteristics and performance measures) are also heavily influenced by how likely a person can succeed at a given option. However, most existing cognitive effort-based research has focused primarily on demand-related factors, leading to confounding inferences about the motivation behind these choices. This study used an adaptive algorithm to adjust relative demand levels for three cognitive tasks to investigate general and individual differences in demand preferences. The results highlight an overall pattern of individual differences in intrinsic motivation to perform challenging tasks, supporting research that found cognitive effort aversive to some but attractive to others. These results suggest that relative demand levels and intrinsic task factors drive the motivation to select an action.

## 1 Introduction

Cognitive effort is a crucial component of all skill-based actions, from those used in work-related environments to the ones we choose when entertaining ourselves. Learning skills involved in playing a musical instrument or even video games come at similar costs as those needed for higher mathematical or scientific endeavors (Kanfer and Ackerman, 1989; Delaney et al., 1998; Haerem and Rau, 2007). However, improving upon any skill requires time and effort, particularly when an obvious payout (fame, money, etc.) is not guaranteed (Debowski et al., 2001; Daw et al., 2006). When studying effort-based decisions, the motivation behind these behaviors becomes more mysterious when considering the constraints. That is, due to limited resources (Rangel et al., 2008; Shenhav et al., 2017) and finite time (Busemeyer and Townsend, 1993; Otto and Daw, 2019), actions are often thought to be weighed by their immediate payout against these factors (i.e., limited time and resources; Botvinick et al., 2009; Lim et al., 2023). This leads to the question of how effortful actions are weighed in these cases when no immediate reward outcome exists.

The current study investigated individual differences in effort-based decisions, specifically focusing on the factors of intrinsic motivation and capability involved in how and when people decide to exert cognitive effort. Intrinsic motivation is defined as the motivation behind acts performed for the sake of performing these actions (Ryan and Deci, 2000), whereas capability is related to the individual’s ability to complete a task (i.e., Westbrook et al., 2013). This article highlights how not separating these two factors can lead to confounding inferences about the motivation behind cognitive effort based decision-making (i.e., Westbrook et al., 2013). Toward this goal, we present a series of experiments that attempt to control for individual differences in capability in order to measure preferences between a task offered at two different (but individually tailored) demand levels. Results suggest that both the ease of an option (as measured by performance) and the relative demand levels (as inferred by the difference between performances in each option) can influence decisions. These results also support the idea that individual differences in intrinsic motivation may be partially explained by the type of task offered. Finally, we discuss the implications of these results for future research on effort-based decision-making, particularly how performing a task itself can add value to an action and motivate decisions.

### 1.1 Previous theoretical work

Theories on how people exert cognitive effort assume that utility functions are used to calculate the value of each option by weighing factors involved in decisions. In these utility-based models, effort costs are weighted by (1) what an action requires (Botvinick et al., 2009; Hélie et al., 2017); (2) individual capability to perform the action (e.g., Kool et al., 2010; Sandra and Otto, 2018); and (3) the motivation to invest effort to obtain a reward (Busemeyer and Townsend, 1993; Bonner, 1999; Bonner and Sprinkle, 2002). Integrating these factors and how they interact with each other leads to predictions about how likely someone is to choose to perform an action. This is due to the interaction of person and task characteristics changing how much “cost” that action demands, leading to differences in motivation to perform a task (Busemeyer and Townsend, 1993). Demand levels are often used to manipulate this likelihood, with differences in effort often measured using task performance (see Wood, 1986; Bonner and Sprinkle, 2002). Performance is an intuitive way to infer the difficulty of a task because it is objective and measurable: if a person shows higher performance in one version of a task compared to another version of that same task, then the difficulty of that first version should be considered lower. However, performance has also been used as a measure of motivation, with the desire not to exert any effort as a reasonable justification for lower performance instead of capability or higher task demand (Sandra and Otto, 2018). While evidence supports that most people prefer lower-demanding options (Botvinick et al., 2009; Kool et al., 2010; Shenhav et al., 2017), these inferences often do not account for the multitude of alternative interpretations that performance measures may have. Specifically, without controlling for differences in capability, the influences of motivation on performance cannot be separated from capability-related factors.

Much of previous research has focused on how demand preferences relate to the effort costs each given option requires (e.g., Shah and Oppenheimer, 2008; Botvinick et al., 2009; Kool et al., 2010; Shenhav et al., 2017). These articles focused on the interaction of person and task characteristics as directly influencing motivation (versus motivations outside of cost-related factors). For instance, Kool et al. (2010) measured demand preferences across six experiments and generally found that people preferred the low-demand option when given the choice between two options associated with higher or lower performances. In one of their experiments, results even suggested an association between indifferences and those who performed better at the task. These results were interpreted as a general lack of motivation to exert effort because options associated with lower performance were thought to drain more resources than those associated with higher performance. The main problem with this interpretation, however, is that there was no indication of the relative differences in demand levels for participants (was performance in the low-demand and high-demand levels different?). While this experiment took care to control for many stimulus-related factors such as color and location, it did not control for differences in capability.

Cognitive capability here refers to the effort costs a participant experiences when performing a task (e.g., Botvinick et al., 2009; Shenhav et al., 2017). This is objectively hard to define as differences in motivation have been shown to affect measurements such as choices and performance both across (e.g., Westbrook et al., 2013) and within (Sandra and Otto, 2018) participant groups. Without separating how motivated individuals are, it is hard to infer what their capability at a given task is because we do not know for sure that participants are trying as hard as they can. This potential confound can lead to misinterpretations of measurements that capture both capability and motivation. This interaction then challenges the interpretation of effort-based decisions being primarily based on effort costs. There is no meaningful way to define participants who perform poorly as either low motivated or, instead, could be highly motivated but simply cannot perform the task? Those who will not and those who cannot might both be drawn toward lower demanding options but for radically different reasons.

There are other theories that focus on the utility of conserving energy while also considering differences in motivation. As motivation can mean many different things, this article defines motivation as the intensity of wanting to complete a goal or action. Motivation used this way can explain how attractive someone finds engaging in an option by using different types of rewards such as money or task characteristics (e.g., Bonner and Sprinkle, 2002; Botvinick et al., 2009). Much like with capability, performance can reflect differences in motivation by introducing goals that focus on the benefits of performing an action (or reward sensitivity), rather than strictly the cost. For example, Westbrook et al. (2013) suggested that capability and reward sensitivity are closely associated by offering task options at different demand levels for different monetary reward amounts. This study sampled different groups of cognitive capability related to age, assuming that older adults typically experience higher cognitive costs (as inferred by lower performance) than their younger counterparts. Using an adjusting reward structure, participants were asked to choose between two demand levels of a task, both offered at the same starting reward amount. The reward for the high-demand option fluctuated after each of six decisions, raising in value if the low-demand option was chosen and lowering in value if the high-demand option was selected. This allowed the researchers to infer motivation from the final reward amount, with higher final amounts reflecting lower sensitivity to external reward because more money was needed to motivate effort exertion (Westbrook et al., 2013). The authors hypothesized that groups with higher cognitive costs (i.e., older adults) would show less sensitivity to reward and found that their results supported this. In other words, those with lower cognitive capabilities (i.e., higher age group) needed larger amounts of reward to increase their effort investments. However, while this study allowed for differences in motivation, the authors could not separate whether participants were more influenced by the cognitive load or the reward, just that these two factors interacted. That is, interpreting performance as reflecting motivation is also confounded by differences in reward sensitivity, as those who are primarily motivated by an external reward cannot be separated from those motivated internally. Were older adults more averse to the high-demand tasks, or were they less motivated by the monetary reward than their younger counterparts?

Another study attempted to tie cognitive capability with reward sensitivity by using performance in two ways: (1) as a way to classify participants based on motivation and (2) as a proxy to measure reward sensitivity (Sandra and Otto, 2018). The reasoning behind this was theorized that performance in the no-reward condition represented differences in intrinsic motivation. That is, participants would perform according to how rewarding performing the task is without needing an external incentive such as money (Sandra and Otto, 2018). Then, by comparing participants’ performance to a reward conditions, the authors asserted that performance differences would reflect how motivating a monetary reward was against an individual basis of motivation to perform the task (Sandra and Otto, 2018). Results supported these ideas by demonstrating that lower-performing individuals in the no reward condition saw greater increases in performance in a reward condition. These same individuals also scored lower on the intrinsic motivation scales than their higher-performing counterparts (Sandra and Otto, 2018). Higher-performing individuals were also not as responsive to an offered reward. However, while this study found a way to use performance as a measure of motivation, this study did not answer whether this motivation is influenced by capability (Kool et al., 2010; Westbrook et al., 2013). That is, are higher capability individuals differently motivated by effort costs and rewards in that they do not find these tasks costly and, therefore, need less external reward to perform the task? Or is it, as these authors suggest, that capability differences are merely reflective of individual differences in finding performing a task rewarding in itself?

### 1.2 Current article

The present study investigated intrinsic motivation in demand preferences while attempting to minimize differences in capability when not offering a monetary reward. The three cognitive tasks were chosen in an attempt to remove potential confounds of the experimental designs in Kool et al. (2010) and Sandra and Otto (2018). Task-switching requires a level of cognitive ability that makes it harder to separate ability-related decisions from effort-based ones. Specifically, are those with higher ability expending less effort and not making more effortful decisions by selecting higher demanding tasks (Kool et al., 2010, 2013), or are the higher ability participants more motivated than their lower performing counterparts (Sandra and Otto, 2018)? Kool and Botvinick (2013) addressed motivational concerns by proposing that their approach to studying effort-based decisions were simpler and depended on subjective ratings of effort. Following this line of reasoning, the current study selected tasks that were simpler than task-switching (Kool et al., 2010, 2013) (the first two only requiring one main cognitive component) and could “level the playing field” for participants who may have superior skills in either motor or memory-related tasks. The third task experiment was selected to compare these simpler designs with a task that required both memory and motor components. Demand levels in this study were individually defined for each participant during an initial adaptive phase by varying task demands until a pre-specified level of accuracy was reached. In contrast, the number of choice selections for the higher demand level was used to measure motivation. Using a two-force-choice paradigm between two demand levels that are individually tailored to each participant, this study measured how often participants selected a high or low-demand option. If people are generally demand-avoidant (i.e., make choices primarily preferring fewer high-demand options), then offering individualized demand options should be related to differences in demand levels and, by extension, reflective of the effects of cognitive costs (Kool et al., 2010). That is, smaller differences in task demand should be related to higher indifferences, and larger differences in task demand should be related to higher demand avoidance, with little to no preferences for the high-demand option. If, however, people are differently motivated, then presenting individualized demand options should be associated with a variety of demand preferences. Across three different experiments, results suggest that differences in intrinsic motivation can drive demand preferences rather than an overall drive to conserve energy. That is, the choices that participants made should reflect motivations other than those strictly related to capability differences These experiments further support the theory that performance may be a byproduct of initial effort investments unrelated to individual performance.

## 2 Experiment 1

This experiment measured the number of high-demand option selections in a two-forced choice paradigm where the options were of the same task with different demand levels. The main hypothesis was that individual differences in demand selection would be present even when the demand levels were tailored to the individuals. Instead of offering the same high- and low-demand levels to every participant (e.g., Kool et al., 2010; Westbrook et al., 2013), this study used an adaptive algorithm to “set” each demand level according to how well each individual performed. Individualized demand levels should reveal more demand avoidant or indifference in that case because each participant should experience similar cognitive costs. However, if capability is not a predictor of demand preferences, then individual differences in demand preferences should be more closely related to intrinsic motivation (e.g., Cacioppo et al., 1984; Ryan and Deci, 2000).

### 2.1 Methods

This experiment measured demand selection when offered a low and a high-demand version of a visual discrimination task [similar to Shiu and Pashler (1995)]. This experiment used a visual discrimination task as a starting point for the adaptive algorithm since this algorithm was primarily constructed for individualized discrimination adjustments. Previous research using a visual discrimination task has shown a relationship between discrimination found that a relationship between discrimination difficulty and effort judgments in that including a motor component (to push a button in making a discrimination distinction) also influenced how effortful participants perceived the task (Turner et al., 2021). This involved using an adaptive staircase algorithm based on the visual discrimination literature (Lu and Dosher, 2013) to adjust the demand levels to each individual so that the low- and high-demand options each had a similar accuracy level across participants, regardless of skill level.

#### 2.1.1 Participants

Forty undergraduate students were recruited from the Purdue University participant pool. Participants gave informed written consent, as approved by the institutional review board of Purdue University, and were granted 1 h of credit toward their completion of Introduction to Psychology courses. Two participants’ data were excluded due to computer error, leaving the final sample to include 38 participants. A *post hoc* power analysis was conducted based on findings from Westbrook et al. (2013), who compared different age groups of participants, cognitive abilities, and choices. They found a robust effect size between these two groups (*N* = 17) in terms of discounting (Cohen’s *d* = 0.38). Our sample size (*N* = 38) allowed for a power of 0.799, with an alpha of 0.05 using this effect size.

#### 2.1.2 Material and Procedure

**Figure 1:**
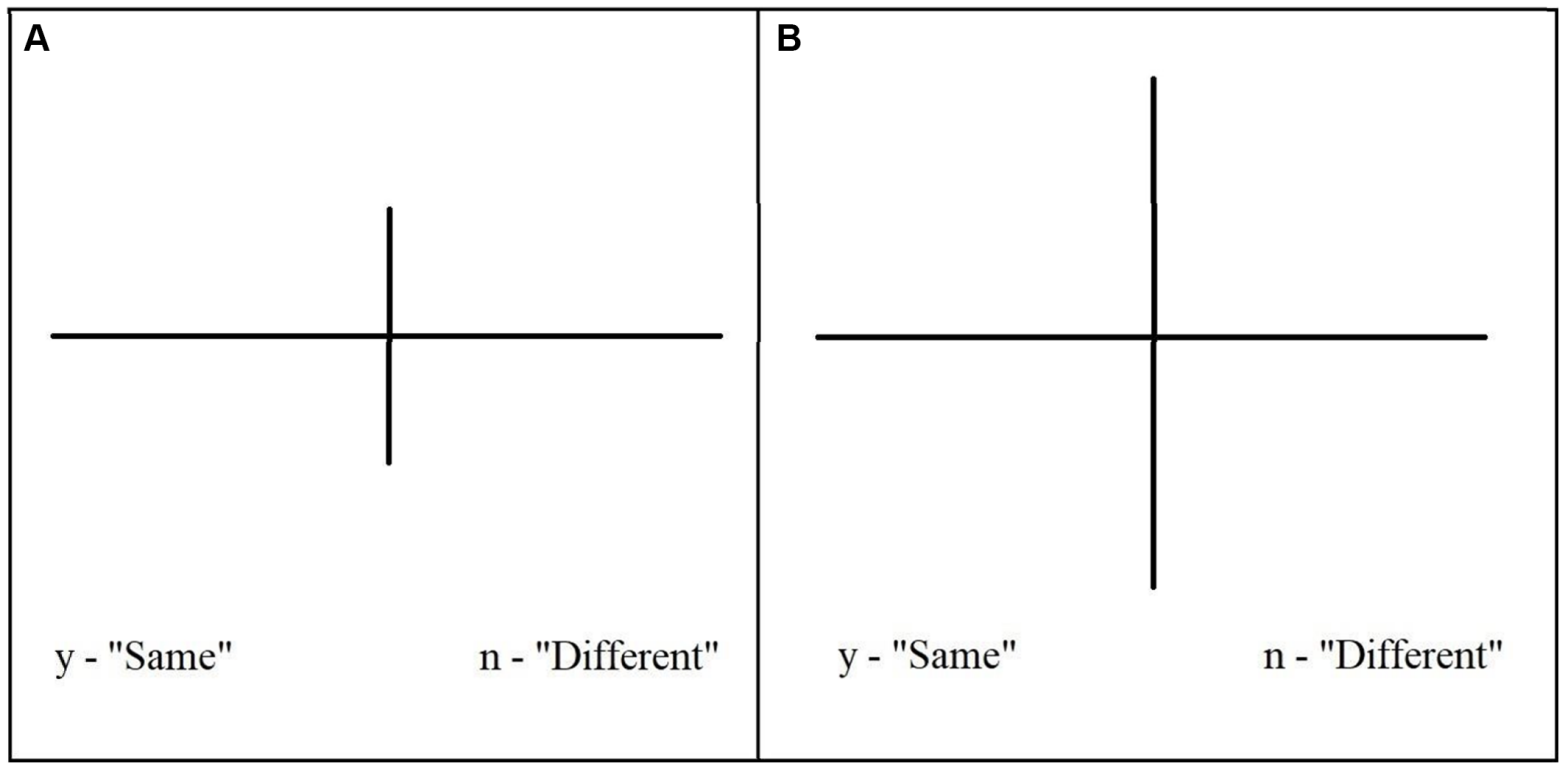
Figure depicts two example trials for a low-demand **(A)** and a high-demand **(B)** version of the visual discrimination task.

The task used in this experiment was computer-based using a Psychopy3 coder (Peirce et al., 2019) and a standard keyboard. In the center of the screen, lines appeared as a cross and were displayed on a 21-inch monitor, with the longest length of both lines reaching a maximum visual angle of 10°. To indicate a judgment, participants were instructed to press “y” to indicate if the two lines were the same and “n” if the lines were different lengths. These keys were marked on the keyboard and indicated above the stimulus at the top of the screen (Figure 1). Participants were given a verbal overview of the task and were informed that they would perform four task phases: training, adaptive, comparison, and choice phase. Each participant was informed that they would be given a choice between two task levels during the choice phase and to “feel free to choose however they wish.”

Once seated at the computers, participants were shown a demonstration on the screen of the task they were to perform. The task required determining whether two perpendicular and intersecting lines on a computer screen were the same or different lengths. Stimuli were presented on the screen for 3 s or until the participants made a response. Participants were required to practice until they achieved 12 correct responses before moving on to the next phase. Accuracy was a correct judgment of whether the two lines were the same or different. Feedback was provided immediately as either “correct” or “incorrect” for 1.5 s before the subsequent trial started automatically.

**Figure 2:**
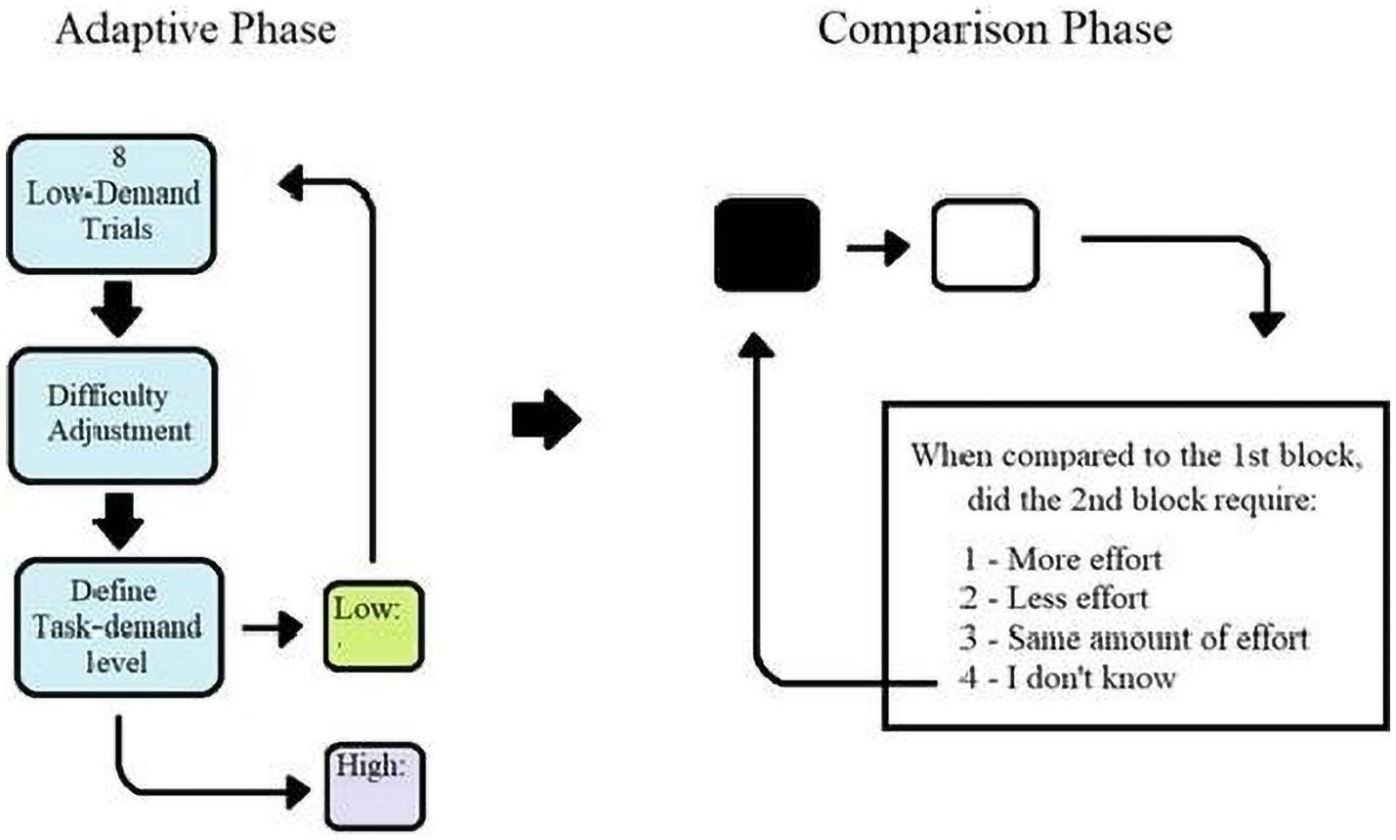
Procedure for all experiments. For Experiment 1, the low – demand was set when accuracy of 95% was achieved and 85% was set for the high – demand. In Experiment 2, the low – demand was set to 90% with the high – demand at 80%. For Experiment 3, the low – demand was set to 75% and the high – demand to 90%.

Participants then entered an adaptive phase (Figure 2), which defined two demand levels based on trial-by-trial accuracy. The algorithm first defined a low-demand level, with the first eight trials starting at the lowest difficulty (with the vertical line being 50% of the horizontal line length). After these eight trials, difficulty increased or decreased depending on accuracy. If participants obtained 12 correct consecutive trials, the vertical line length percentage increased by 3.5%, with the maximum difficulty containing up to 90% of the horizontal line length. If participants made an error, the difficulty decreased by 3.5%, with the minimum being 50% of the line length. Once participants reached below 90% overall accuracy, the line length was used to define the low-demand level for that participant. After the low-demand level was defined, the adaptive algorithm was repeated, starting with eight trials at the lowest difficulty, and demand increased or decreased with the same criteria until participants reached a response accuracy under 80%. At that point, the high-demand level was defined.

The two demand levels were randomly assigned to either a “White” or “Black” condition. Participants performed two blocks of eight trials with each demand level, with the order of blocks randomly assigned and each block referred to by their assigned color. After these blocks, participants were asked to rate whether the second block was “less effortful,” “more effortful,” had the “same amount of effort,” or “I do not know.” If participants selected “I do not know,” they performed the eight trials of each demand level again up to three times, after which the experiment continued to the choice phase. None of the participants had to repeat this section more than once.

Finally, participants entered a choice phase consisting of 24 blocks with 10 trials in each block. Each trial was worth one point in both demand levels for these trials. Before the beginning of each block, participants were informed of the number of points they received in the previous block with that demand level (e.g., if presented with a white block, participants would see “White trials, your last score in white was ___”). For 18 blocks, participants were allowed to choose freely between the White and Black conditions, with the other six being pre-selected for them (trials: 1, 2, 12, 13, 23, 24). This was done to establish a baseline accuracy for exploratory analysis in cases where a participant only chose one demand level. At the end of the choice phase, participants were thanked for their time and instructed on-screen to exit the experiment room for debriefing.

#### 2.1.3 Data analysis

The main part of this analysis focused on the number of high demand options selected in the 18 choice blocks within each participant. This number was then used to categorize participants as (1) demand avoidant (those who selected the high-demand option 4 or fewer times out of 18 decisions), (2) challenge-seeker (those who selected the high-demand 14 or more times), or (3) indifferent (everyone else). This criterion was selected based on past research, which found that about 20% of behaviors in a new low-reward context can be interpreted as random exploration (Dezza et al., 2017).

An exploratory analysis compared these categories with individual differences in capability. While each level was tailored to the individual, performance and demand levels both can reflect differences in capability. This study attempted to set a similar performance level in order to try to control the amount of effort in each option. However, participants typically vary in how much demand levels are needed to reach, say, at 90% performance rate. As such, this analysis explored whether or not differences in demand levels can explain differences among categories of participants.

### 2.2 Results

#### 2.2.1 Subjective rating of effort and manipulation check

Subjective ratings of effort were analyzed. A congruent rating considered the high-demand option more effortful than the low-demand option; an incongruent rating considered the low-demand option more effortful, and an indifferent rating indicated no subjective difference. Subjective comparison of these options suggests that most participants (N = 29) viewed these two as distinct in that they rated the high-demanding option more effortful than the low-demanding option. One participant reported the low-demand option as more effortful, and 8 participants reported they could not distinguish between them.

Manipulation check of performance was performed on trials during the exposure phase. A paired sample *t*-test of the low versus high options resulted in two statistically different demand levels (*t*(37) = 6.801, *p* < 0.01, CI 95% (0.071, 0.130), with the low-demand option associated with higher performance (*M* = 94.26, SE = 0.018) than the high-demand option (*M* = 84.21, SE = 0.023).

#### 2.2.2 Demand preferences

**Figure 3:**
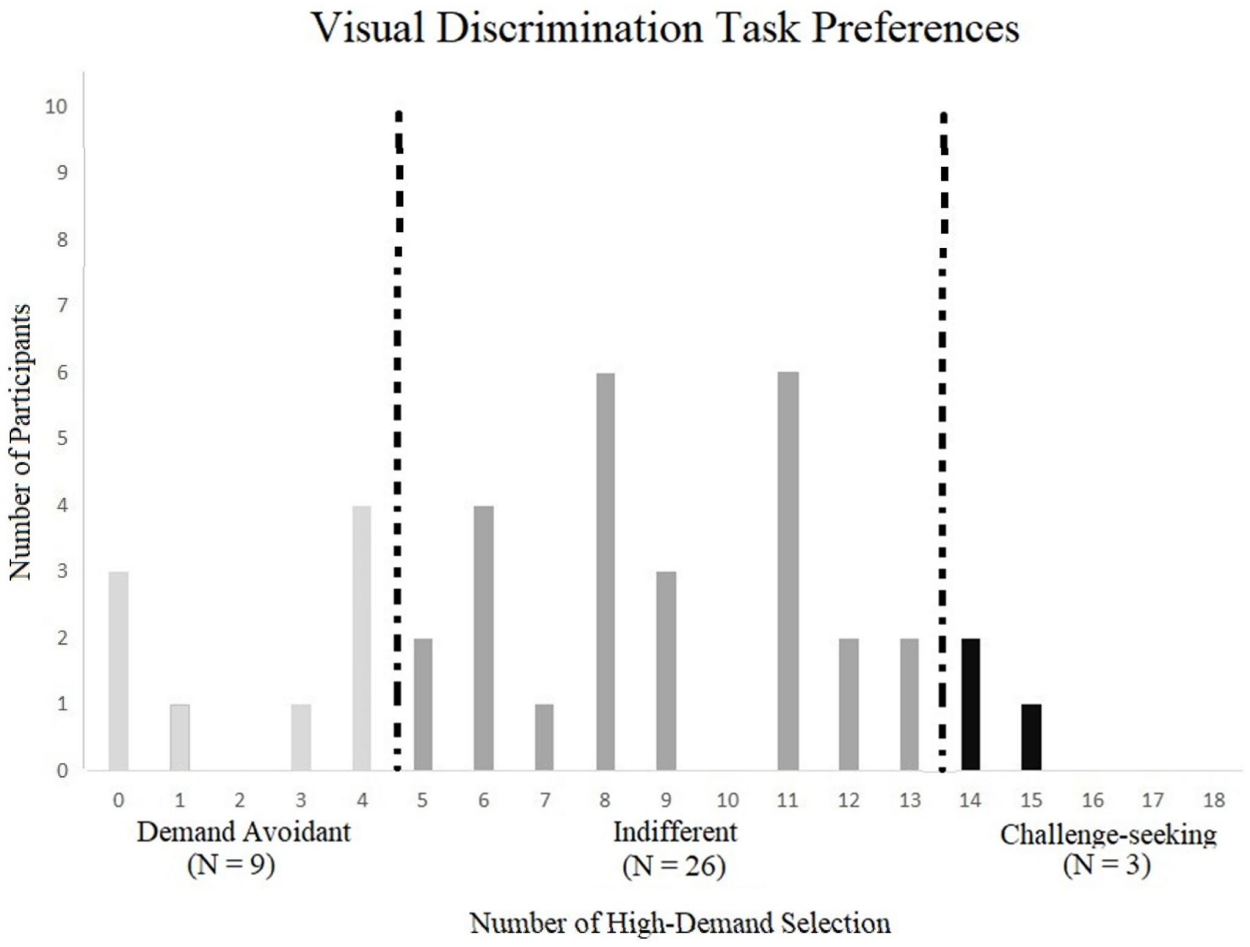
Demand preference categories by number of high – demand selection in the visual discrimination task.

The number of high-demand choices made during the choice phase, ratings of cognitive effort, and performance (i.e., accuracy) were recorded to determine the relationship between capability and preferences. The number of high-demand choices was compared descriptively for each participant and as an overall average for group choices to measure demand preferences. Nine out of 38 participants indicated demand avoidance and three preferred the higher-demanding task, leaving 26 as indifferent (Figure 3).

To ensure that this distribution of preferences was related to experimental design and not random chance, these results were compared to a simulation of choices to create a random distribution. This involved simulating 18 decisions (randomly choosing low or high demand with probability *p* = 0.5) for each of the 38 simulated participants. This simulation was run 10,000 times to create distributions of participants who would fall into the avoidant, indifferent, or challenge-seeking categories by chance in 10,000 simulated experiments. For the avoidant category, the average number of simulated participants was under one per simulated experiment (*M* = 0.590, CI 95% [0.575, 0.605]). For the challenge-seeking category, the average number of simulated participants was similarly under one per simulated experiment (*M* = 0.587, CI 95% [0.575, 0.605]). Finally, the indifference category had the highest number of simulated participants in each simulated experiment (*M* = 36.823, CI 95% [36.802, 36.844]).

#### 2.2.3 Capability and demand levels

To investigate whether categories of participants represented differences in capability, these groups’ demand levels were compared (i.e., how long were the low-demand and high-demand lines to achieve the desired accuracy). Since very few participants showed a preference, the average demand level in the indifferent group was compared against the average demand levels for the other two groups. There were six available levels, each representing a difference in vertical line length of 3.5% of the horizontal line. The indifferent group (*N* = 26) had an overall numerically lower average vertical line length than the other two groups for both the low demand (*M* = 0.562, CI 95% [0.556, 0.568]) and high demand (*M* = 0.688, CI 95% [0.672, 0.705]) levels. However, the average for the demand avoidance participants (*N* = 9) was within the 95% confidence interval of the indifferent participants for both the low demand (*M* = 0.566) and high demand (*M* = 0.699) levels. The challenge seeker group was outside this range, but just barely for the low demand (*M* = 0.570) and high demand (*M* = 0.710) levels. This suggests that challenge seekers may have had slightly higher capacity in this task. In addition, a Pearson’s correlation was computed using the number of high-demand choices and accuracy in the high-demand level and was not statistically significant (*r* = 0.195, *p* = 0.240, CI 95%[0.485, 0.132]).

### 2.3 Discussion

This experiment measured demand preferences between high- and low-demand versions of a visual discrimination task while using an adaptive algorithm to personalize the demand options. This allowed for the investigation of capability in demand preferences by presenting two options with similar demand levels across participants. Results suggested that there were individual differences in preferences that were statistically different than chance. When comparing demand levels across categories of participants, these levels were similar between the indifferent group and the demand avoidant groups, suggesting that individual differences in demand preferences may be more closely related to differences in intrinsic. However, there was a difference between the challenge-seeking group and the other two in that these participants, on average, had higher demand levels. These results support the relationship between capability and intrinsic motivation. However, due to the low sample size of the challenge-seeking group, further investigation is needed. Overall, these results challenge utility-based theories which suggest that these individual differences were due to differences in capability. That is, rather than individual differences being associated with how capable a person is at performing a given option, these participants’ demand preferences are consistent with differences in motivation.

## 3 Experiment 2

The results of Experiment 1 show that previous research suggesting that people are primarily demand-avoidant may have been strongly influenced by differences in capability. Individual differences remained even with the task levels adjusted to each participant’s skill level: Some participants were demand-avoidant while others were challenge-seeking. Experiment 2 further explored the influence of capability and intrinsic motivation by using a motor task and making both demand levels of the task more effortful. If the relative difficulty of demand levels in the options determined task selection, then results should indicate a similar indifference in this study. This should be prevalent even when increasing the absolute demand levels of both options. However, changing the perceptual task to a motor task may also affect individual choice differences if choices reflect intrinsic task characteristics.

### 3.1 Methods

This experiment was similar in design to Experiment 1, with the training, adaptive algorithm, comparison, and choice phases being presented in the same order and presentation. This study used a motor-interception task (described below), and performance was similarly used to set demand levels.

#### 3.1.1 Participants

Forty new undergraduate students were recruited from the Purdue University participant pool.

Participants gave informed written consent, as approved by the institutional review board of Purdue University, and were granted two credits toward their completion of introduction to Psychology course. Two participants exited the experiment early and were excluded from the final analyses.

#### 3.1.2 Materials and procedures

**Figure 4:**
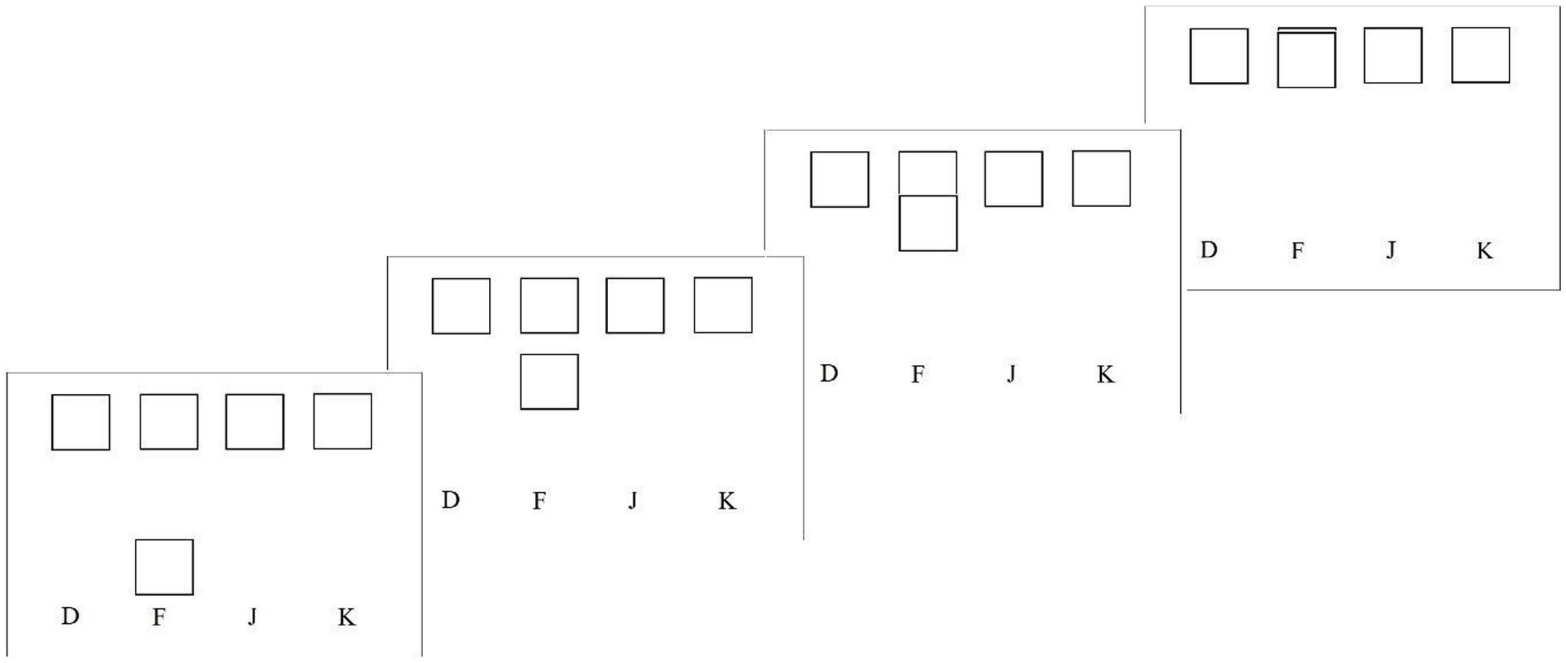
Example trial for the motor – perceptual task.

Procedures were identical to those in Experiment 1 except for the task to be performed. This experiment used a motor interception task [similar to Sanchez et al. (2010)] in which four target squares were presented at the top of the screen across a visual angle width of 34.71° by a height of 2°. Each square was associated, respectively, with the letters (“d,” “f,” “j,” k”) indicated at the bottom of the screen and marked on the keyboard (Figure 4). At the start of each trial, a fifth square would appear above a letter (which letter was randomized), moving upward. When the moving box intercepted the target square at the top of the screen, participants were instructed to press the key associated with that square (the letter directly below the square). The speed of the moving square varied depending on participants’ performances and was used to manipulate task demand. Task demand levels were set at 80% for the low-demand option and 70% for the high-demand option to keep the relative difference between the levels similar to Experiment 1 but more challenging overall.

At the start of the experiment, participants were given verbal instructions and shown a demonstration of a successful trial. Each trial lasted 3 s with a one-second intertrial interval (ITI). Feedback was given immediately after the response at the top of the screen as either “correct” or “incorrect” throughout the ITI until the next trial began. The training phase required participants to achieve accuracy on at least 10 trials, with accuracy being defined as pressing the correct key in combination with at least 50% overlap between moving and target squares. The trial was marked as incorrect if a key was pressed when the box did not overlap. In the adaptive phase, the speed of the moving square began at 0.35 pixels/frame using a 21″ monitor with 1,920 × 1,080 resolution with a 60 Hz frame rate. It was adjusted by 0.05 pixels/frame, with the slowest speed being 0.25 pixels/frame and the fastest speed being 1.15 pixels/frame.

Participants were then shown each level, as in Experiment 1, and were asked to make a subjective rating of how one block compared in effort toward another. After this, participants were asked to choose between these two options 18 times.

### 3.2 Results

#### 3.2.1 Subjective rating of effort and manipulation check

Subjective ratings indicated that 29 out of 38 participants rated the high-demand option as more effortful than the low-demand option. However, 6 participants rated the low-demand option as more effortful, suggesting further nuances in participant effort ratings and object measurements (Kurzban et al., 2013). One participant indicated they could not distinguish between the two demand levels.

A manipulation check for this experiment used the performance during the exposure phase, which suggested two statistically distinct levels of demand (*t*(37) = 20.513, *p* < 0.001, CI 95% [23.146, 27.001]). The high-demand option had lower accuracy (*M* = 0.5654, SE = 0.0151) than the low-demand option (*M* = 0.80111, SE = 0.0151). The accuracy difference between the demand levels was similar to Experiment 1, but the overall demand was higher, as inferred by the lower accuracy.

#### 3.2.2 Demand preferences

**Figure 5:**
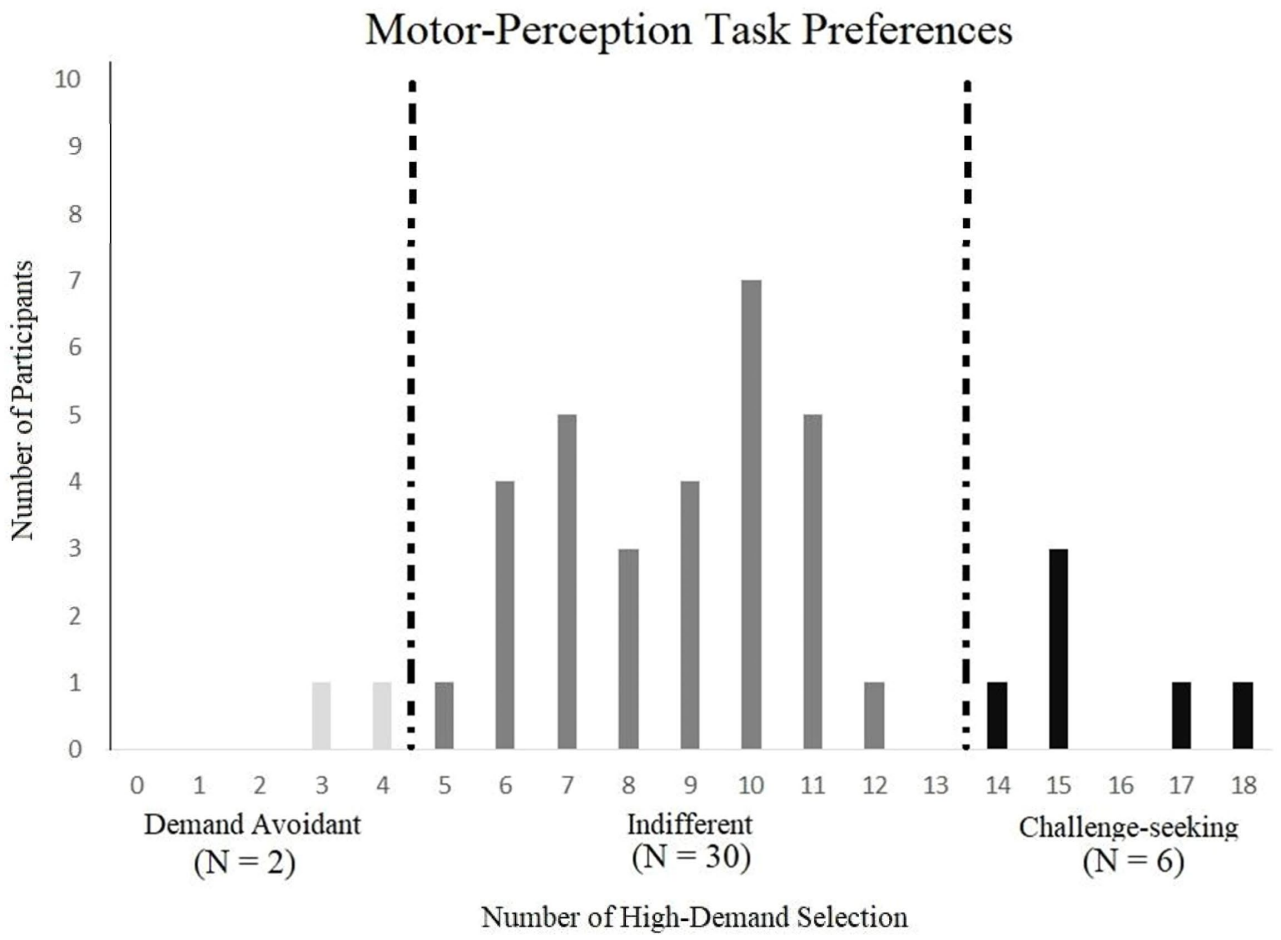
Demand preference categories by number of high – demand selection in the motor perceptual.

The number of high-demand selections was used to categorize participants as either demand-avoidant (i.e., participants chose the high-demand option four or fewer times), challenge-seeking (participants chose the high-demand option 14 or more times), or indifferent. Two participants were categorized as demand-avoidant, six were labeled challenge-seeking, and the remaining 30 were indifferent (Figure 5). A Pearson’s correlation was computed using the number of high-demand choices and accuracy in the high-demand level and was not statistically significant (*r* = 0.265, *p* = 0.108, CI 95% [−0.060, 0.539]).These results were compared to a simulation (as shown in Experiment 1 with a similar sample size) and showed that the obtained distribution was statistically different from chance.

#### 3.2.3 Capability and demand levels

The confidence interval of demand levels was taken for the indifferent group and used as a comparison point of capability across the different categories of participants. There were six available demand levels, each representing a difference in pixel/s frame of 0.05. The indifferent group (*N* = 30) in the low demand (*M* = 0.583, CI 95% [0.565, 0.601]) had higher accuracy than the demand avoidant group (*M* = 0.55) and the challenge-seeking group (*M* = 0.533). There were no differences among the groups seen when comparing the indifferent group in the high demand (*M* = 0.818, CI 95% [0.774, 0.460]) in that the demand levels were similar to the demand avoidant group (*M* = 0.55) and the challenge-seeking group (*M* = 0.525). Overall, the indifferent group had a higher capacity, but only in the low-demand condition.

### 3.3 Discussion

Like Experiment 1, the results of this study indicated individual differences in demand preferences, suggesting that there may still be an intrinsic motivation that influences different types of people. These choices were statistically different than chance, indicating that the motor-interception task similarly tapped into demand preferences as the visual discrimination task. Demand levels were again used to infer differences in capability and found little to no differences among the categories of participants. The only difference observed was for the low-demand option, with indifferent participants showing higher capability. Lastly, the subjective ratings suggested that the perception of task demand may be more nuanced than initially thought. The incongruent ratings could be due to the subjective nature of whether accuracy for a faster-moving object is more effortful than a slower moving object. However, this was not reflected in their performance (i.e., higher demand was always associated with lower performance than the low-demand option).

## 4 Experiment 3

The results of Experiments 1 and 2 suggest that demand preferences are individually based even when controlling for differences in ability and across two separate tasks. Experiment 3 furthered the investigation of relative demand preferences by offering two components: (1) a higher relative difficulty difference between the demand options and (2) a working-memory cognitive task component. Working memory is cognitively demanding, with more items to manipulate associated with higher demands (e.g., Yee and Braver, 2018). If demand preference reflects the desire to conserve energy, then participants should favor the low-demand option. However, cognitive challenges can be appealing to some participants (Cacioppo et al., 1984), and differences in demand preferences reflect differences in intrinsic motivation (Ryan and Deci, 2000). In that case, demand preferences should be more polarized, with fewer participants showing indifference.

### 4.1 Methods

#### 4.1.1 Participants

Using the Purdue University participant pool, 40 new undergraduate students were recruited.

Participants gave informed written consent, as approved by the institutional review board of Purdue University, and were granted 1 h of credit toward their completion of Introduction to Psychology course.

#### 4.1.2 Materials and procedures

Experiment 3 used the same phase order, procedure, and materials as those used in Experiment 2. This Experiment involved a training, adaptive, comparison, and choice phase while using a working memory/motor task. During the task, four black lines occupied a visual angle width of 12.5° by a height of 18.9° across a 21-inch monitor (1,920 × 1,080 resolution). The stimuli included white blocks, each occupying an approximate visual angle of 2°. Finally, participants were required to use the keys “d,” f,” j,” and “k” on a standard keyboard.

**Figure 6:**
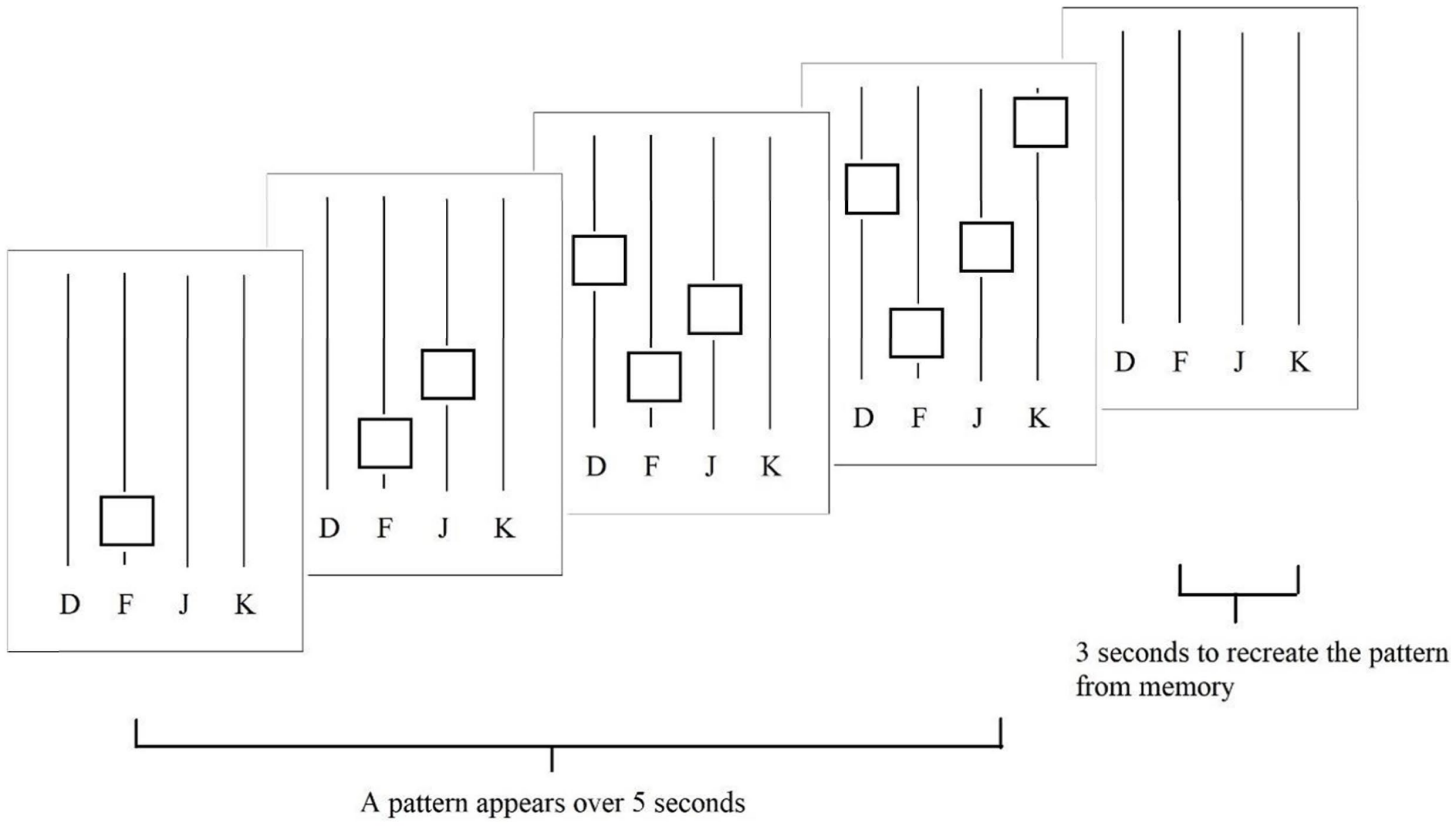
Example trial for the working memory/motor task.

Participants were given verbal instructions as well as shown an example that these white boxes would appear on one of the four lines one at a time from the bottom to the top of the screen (Figure 6). When the boxes disappeared, they were to recreate the pattern using the keys associated with the line in the order that the boxes appeared (similar to playing the piano). For the first 5 s of the trial, a pattern of white boxes appeared on the screen and then disappeared, and for the remaining 3 s of the trial, the participants only saw the four white lines with their associated keys. Accuracy was considered a complete recreation within a 3 s timeframe, and participants received feedback on a correct or incorrect trial on the screen above the four lines at the start of the subsequent trial for 1.5 s ITI.

For the adaptive phase, the increase or decrease in difficulty was plus or minus one square, with the lowest demand being four squares and the highest demand up to ten. To establish two distinct demand levels, the low-demand level was defined after reaching 90% accuracy, and the high-demand level was defined below 75%. The procedure did not change for the comparison and choice phases.

As in the first two experiments, participants were then shown a block of each level and asked to compare how effortful they were in relation to each other. During the choice phase, participants were then given 18 chances to choose between these two options.

### 4.2 Results

#### 4.2.1 Subjective rating of effort and manipulation check

Performance during the exposure phase suggested two statistically distinct levels (*t*(39) = 9.526, *p* < 0.001), with the high-demand showing lower accuracy (*M* = 0.661, SE = 0.299) than the low-demand option (*M* = 0.91, SE = 0.167). Subjective ratings suggested that these two levels were subjectively distinct in that all 40 participants rated the high-demand option as more effortful than the low demand option. The high-demand accuracy was lower than the initial aim due to the task difficulty increasing drastically when adding a square. However, this lower accuracy was seen across all participants and still applies to the aims of this experiment.

#### 4.2.2 Demand preferences

**Figure 7:**
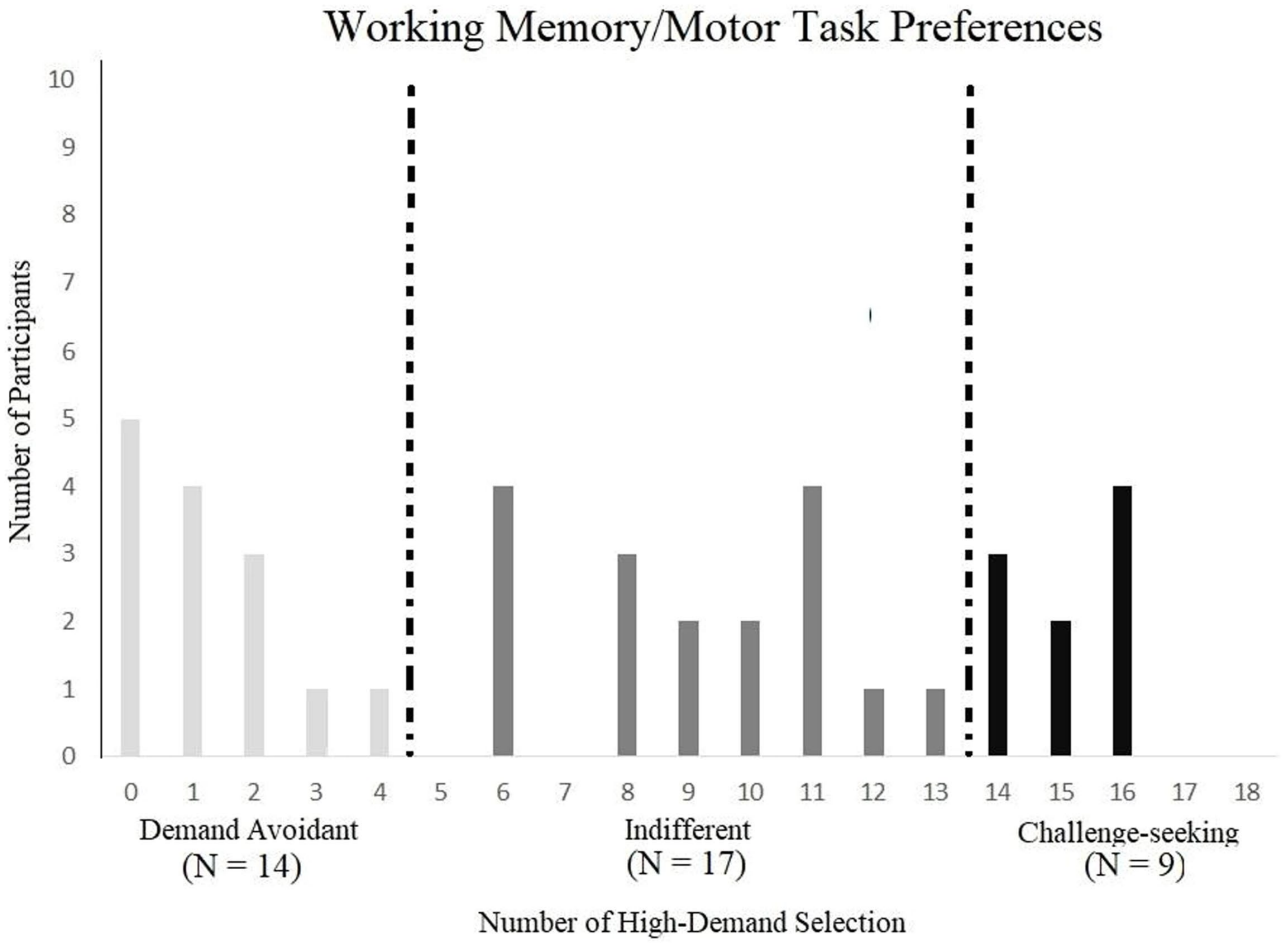
Demand preference categories by number of high – demand selection in the working memory/motor task.

Fourteen participants demonstrated demand avoidance, 9 demonstrated challenge-seeking behaviors, leaving 17 indifferent (Figure 7). We used a similar simulation to Experiments 1 and 2 to bootstrap a random distribution with a sample size of 40 simulated participants and found that each group was statistically different than chance. The chance simulation predicted the following distribution: number of avoidant participants (*M* = 0.626, CI 95% [0.611, 0.642]), number of indifferent participants (*M* = 36.823, CI 95% [38.723, 38.766]), and number of challenge-seeking participants (*M* = 0.629, CI 95% [0.614, 0.644]). Finally, a Pearson’s correlation was computed using the number of high-demand choices and accuracy in the high-demand level and was not statistically significant (*r* = 0.282, *p* = 0.078, CI 95%[−0.033, 0.545]).

#### 4.2.3 Capability and demand levels

The confidence interval of demand levels was again taken for the indifferent group and used as a comparison point of capability across the different categories of participants. There were six available demand levels, each representing the number of squares. The indifferent group in the low demand (*M* = 4.235, CI 95% [4.010, 4.460]) had a larger number of blocks than the demand avoidant group (*M* = 4.000) but did not appear to be different than the challenge-seeking group (*M* = 4.333). This trend was also seen when comparing the indifferent group in the high demand (*M* = 6.647, CI 95% [6.019, 7.275]) in that the demand levels were higher than the demand avoidant (*M* = 5.929) but not different than the challenge-seeking group (*M* = 6.444). Overall, demand-avoidant participants seem to have a lower capability compared to the indifferent group.

### 4.3 Discussion

Overall, Experiment 3 showed less indifference and a larger number of participants classified as demand-avoidant and challenge-seeking compared to Experiments 1 and 2. When comparing demand levels across categories, the indifferent group had a higher capability than the demand-avoidant group in both the low and high demand levels. These results support the theory of intrinsic motivation, suggesting that different people may value higher demand levels as either a cost (in the case of the demand avoidant) or a reward (for challenge-seekers). That is, when the demand levels of the options offered were more distinct, participants indicated more polarized preferences.

### 4.4 Exploratory comparison across experiments

The results of the first three experiments’ participant distributions were compared using a Bonferroni correction for multiple testing. Because each data set was used for two tests, *p*-values <0.025 were considered statistically significant after correction. The distributions of participant preferences in Experiments 1 and 3 were not significantly distinct from each other (even without multiple-testing correction; *X*^2^ (2, *N* = 78) = 5.94, *p* = 0.051), whereas the distributions of participant preferences in Experiments 2 and 3 were statistically different (*X*^2^ (2, *N* = 78) = 11.42, *p* < 0.01). The difference in distribution of participant preferences between Experiments 1 and 2 did not survive correction for multiple testing (X^2^ (2, N = 78) = 7.27, *p* = 0.03). These results were primarily driven by the demand avoidant and indifferent categories in that Experiment 3 had a larger number of demand-avoidant participants (*N* = 14), and a lower number of indifferent participants (*N* = 20) when compared to Experiment 2’s demand-avoidant (*N* = 2) and indifferent (*N* = 31) categories. These findings could be due to either (1) differences in cognitive tasks or (2) relative differences between demand levels used in these experiments. However, the lack of significant findings between Experiments 1 and 3 makes these results harder to interpret. Further investigation would be needed to separate the possibility that these results are due to the presented demand levels or the tasks themselves. Another possibility is a smaller effect size for the perceptual manipulation (Experiment 1) than for the motor manipulation (Experiment 2).

## 5 General discussion

This series of experiments investigated demand preferences when accounting for individual differences in skill level, allowing for a comparison of effort investments using different cognitive task options. Experiment 1 used a visual discrimination task and offered two demand levels that were statistically distinct but still relatively close to each other. While demand preferences are often associated with performance and the desire to conserve energy, participants indicated a relative indifference toward the demand levels, with some indicating a preference for either the high or the low-demand option. These findings suggest two ideas: (1) demand preferences may be related to how distinct the options are from one another, with similar options leading to indifference, and (2) there are individual differences in motivation to invest effort that are not overtly related to challenge valuations (as indicated by different participants making different choices). These results were further replicated in Experiment 2 using a motor interception task. Together, these results challenge the theory that tasks are primarily preferred based on their cognitive costs (Kool et al., 2010) or performance in that task (Bandura, 1974).

Experiment 3 further supported these differences by offering a cognitively demanding task with two demand levels set to be more distinct from each other (compared to Experiments 1 and 2). These differences appeared to be associated with larger differences in demand preferences. While these individual differences are hard to separate from the task change or the demand level distinction, these results support that overall cognitive investments arise from an interaction of intrinsic motivations and demand options. That is, even prevailing theories involving intrinsic motivation relate increases in choosing a more demanding task as related only to the task demands (e.g., Cacioppo et al., 1984). However, there may be factors intrinsic to the task in ways not related to how effortful these tasks are that spark differences in intrinsic motivation. That is, not all participants will make the same choices given a specific task tailored to one’s capacity. However, to say that individuals make decisions completely unrelated to capability is not supported by the present results. Specifically, increasing the task demands by adding more cognitive components or making the high-demand level more challenging than the low-demand level only accentuated individual demand preference differences in that more participants favored or avoided demanding options. These results do support the idea that task types, relative demand levels of options presented, and capability-related factors all interact to influence cognitive effort-based decision-making. Perhaps acting as a word of caution when designing future experiments attempting to further the investigation into different types of motivations behind these decisions.

### 5.1 Differences in motivation

Individual differences in intrinsic motivation, or the desire to act without an external payout, may be related to the task’s challenge (Cacioppo et al., 1984). As to why a challenge would be motivating, there are many thoughts. A possibility is that people who seem more internally driven to pursue challenging options consider that these effort investments provide opportunities for personal growth (Ryan and Deci, 2000). Past research investigating the relationship between overall life satisfaction and motivation found that motivation is often related to different outcomes. People who appear to be more motivated by external rewards such as money and social praise differ from those who enjoy pursuing challenging tasks for the sake of the challenge (also see Pink, 2011). These internal rewards are often contrasted with external rewards, with the idea that internal rewards can lead to long-term satisfaction in place of an immediate payout. This is seen in research that often associates external rewards with lower life satisfaction because there is a sense of being unable to satisfy these individuals, and there is always another reward to gain (Pink, 2011).

While the intrinsic desire to perform a challenging action may initially conflict with an energy conservation theory, these preferences may reflect differences in valuing distal goals (Hélie et al., 2017). That is, actions can be categorized as either explorative in that these behaviors can increase information, or actions can be exploitative in that an external reward such as money and points are needed to incentivize effort costs (e.g., Gupta et al., 2006). Explorative behaviors are still utility costs but ones that are not focused on immediate results (Daw et al., 2006). They are about gambling the cost of performing an action against the risk or not acquiring an available reward to learn new behaviors that may lead to better rewards later (e.g., Jarvis et al., 2022). Challenge-seeking behaviors may immediately be more demanding, but they have the advantage of possibly leading to strategies that can increase energy savings overall, such as acquiring information or refining skills (e.g., Kanfer and Ackerman, 1989; Haerem and Rau, 2007). Performing an intellectual challenge can be rewarding because one gains information and can increase skills.

Therefore, a strong possibility is that individuals with higher intrinsic motivation may seek challenges to improve mental capabilities and skills (Kanfer and Ackerman, 1989; Daw et al., 2006). That is, instead of relating motivation type with individual states (such as happiness and/or implied personality differences), the motivation to perform challenging events may be to specifically increase capability. That is, capability can explain effort-based decisions by varying the amount of effort one has to initially invest (Bonner and Sprinkle, 2002). However, the continued investment of effort once capability and immediate outcomes are known still remains to be investigated. Results from this study suggest that part of this answer may lay in the task options being presented because different tasks had different distributions of preferences that appeared across different types of tasks.

The reason that capability may influence decisions can be inferred by decision-making research into heuristics, or the strategies people create to make faster decisions (Payne et al., 1993; Shah and Oppenheimer, 2008). These heuristics were theorized to be formed using available cues from past and current experiences and used for updating the value of these cues based on the ensuing outcome of a selected (and performed) action (Payne et al., 1993). For example, one way to investigate these strategy formations is by using a computationally intractable problem or one that needs an unreasonable amount of time and effort to process every available piece of information in a problem (Pizlo and Stefanov, 2013). Results indicated that participants could find near-optimal solutions quickly in such a paradigm, suggesting that these decisions used shortcuts. This interpretation was further supported by the authors’ computational models which used a hierarchical clustering structure similar to how the visual system processes incoming information (Pizlo and Stefanov, 2013). In this light, individual differences in demand avoidance may reflect these different strategies, with some individuals choosing a higher demanding option if they find this effort may instead save computational processing of making decisions.

### 5.2 Limitations and future research directions

There is room when interpreting why challenge-seeking behaviors may be intrinsically motivating beyond the desire for personal improvement. For instance, Otto and Daw (2019) offered a series of choices that manipulated cognitive and physical effort tasks as well as the duration of a task. They found that decisions depended mainly on duration rather than the type of effort in that people preferred to conserve time rather than effort. They further suggested that decisions involving time could be related to whether or not the person thought a given activity was the optimal action they could be performing at that moment (Otto and Daw, 2019). In this light, aversion toward low-demand options could reflect a loss or opportunity cost in that performing a high-demanding option could lead to improved skills at the task and might be viewed as more worth the time spent.

In addition, one major limitation of this study is the smaller sample sizes for each experiment. This study conducted a *post hoc* power analysis based on Westbrook et al. (2013) and indicated a power of 0.799. However, this effect in Westbrook et al. (2013) was a smaller effect of individual differences and a replication study of 300 participants only found half this effect size (Kramer et al., 2021).^1^ The effects found in this study, however, do appear to be replicated across the four experiments even with this limitation but future research based on this work should take care to pre-register a power analysis *a priori* and pay careful attention to cross experimental analysis and inference.

Future research should compare task options against other conditions that may be aversive for reasons unrelated to their demand preferences. For instance, evidence supports that doing nothing is aversive and that participants prefer negative stimuli to sitting in a quiet room (Wilson et al., 2014). A way to investigate whether seeking out more relatively demanding options could be to compare a task against a no-task condition. Another factor impacting decisions could be different cognitive demands, such as those requiring memory components or tasks that combine fine motor skills and cognitive capability to investigate whether preferences are related to tasks or performance. Finally, as this type of research concerns subjective motivations, future experiments should include related measurements such as the Need For Cognition scale (Cacioppo et al., 1984), which measures levels of intrinsic motivation for cognitively challenging tasks. While this study did measure participants’ ability, given that it is a new experimental task design, additional scales for measuring cognitive ability, such as the Trail Making Test (Arnett et al., 1995) should be included to measure working memory, task switching, and visual attention to relate these experimental tasks to other effects found in previous literature.

### 5.3 Conclusion

Cognitive effort investments can reveal individual differences in intrinsic motivation. Our studies showed that accounting for differences in capability by providing relatively demanding options can lead to a wider variety of demand preferences. These findings challenge the assumption that high demand options are aversive and suggest that previous demand avoidance may be due to skill level rather than a preference to avoid exerting effort.

## Data availability statement

The datasets presented in this study can be found in online repositories. The names of the repository/repositories and accession number(s) can be found at: https://osf.io/dwqcx/?view_only=79d7e46c69c046eba511141a2f583845.

## Ethics statement

The studies involving humans were approved by Purdue University’s Institutional Review Board. The studies were conducted in accordance with the local legislation and institutional requirements. The participants provided their written informed consent to participate in this study.

## Author contributions

AR: Conceptualization, Data curation, Formal analysis, Investigation, Methodology, Project administration, Software, Validation, Visualization, Writing – original draft. SH: Conceptualization, Funding acquisition, Resources, Supervision, Validation, Writing – review & editing.

## Footnotes

^1^We would like to thank an anonymous reviewer for making us aware of the Kramer et al. (2021) article.
